# A thermodynamic study on relationship between gas separation properties and microstructure of polyurethane membranes

**DOI:** 10.1038/s41598-023-32908-7

**Published:** 2023-04-13

**Authors:** Mohammad Sajad Sepehri Sadeghian, Ahmadreza Raisi

**Affiliations:** Department of Chemical Engineering, Amirkabir University of Technology (Tehran Polytechnic), Hafez Ave., P.O. Box 15875-4413, Tehran, Iran

**Keywords:** Chemical engineering, Statistical physics, thermodynamics and nonlinear dynamics

## Abstract

The lattice fluid (LF) thermodynamic model and extended Vrentas’ free-volume (E-VSD) theory were coupled to study the gas separation properties of the linear thermoplastic polyurethane (TPU) membranes with different chemical structures by analyzing their microstructures. A set of characteristic parameters were extracted using the repeating unit of the TPU samples and led to prediction of reliable polymer densities (AARD < 6%) and gas solubilities. The viscoelastic parameters, which were obtained from the DMTA analysis, were also estimated the gas diffusion vs. temperature, precisely. The degree of microphase mixing based on the DSC analysis was in order: TPU-1 (4.84 wt%) < TPU-2 (14.16 wt%) < TPU-3 (19.92 wt%). It was found that the TPU-1 membrane had the highest degree of crystallinity, but showed higher gas solubilities and permeabilities because this membrane has the least degree of microphase mixing. These values, in combination with the gas permeation results, showed that the content of the hard segment along with the degree of microphase mixing and other microstructural parameters like crystallinity were the determinative parameters.

## Introduction

Membranes have increasingly been used to separate liquid and gas phase mixtures because of their high efficiency, tunable properties and low processing cost^1^. Nowadays, using various kinds of polymers, new membranes have been fabricated with inherent and unique properties^2^. Since the use of the membrane processes is considered due to environmental problems in many sections, including oil and gas industries to separate pollutants such as carbon dioxide, nitrous oxide, and dihydrogen sulfide, more attention is paid to membranes with a dense structure that can notably separate angstrom-sized molecules^3,4^. Polymers with a good affinity to gas molecules and enough available free volume are good candidates for the gas separation membranes^1^. One of the known polymers that have chemically active groups concerning polar gas molecules is polymers with ether oxygen, like polyethers^5,6^. A class of copolymers that contain ethereal bonds and has recently been used for the gas separation applications, is thermoplastic polyurethanes (TPUs)^7^. Microphase separation between their soft and hard segments makes TPU copolymers a good material for the gas separation membranes because this phenomenon leads to higher gas permeability^8^. The structural parameters, like hard/soft segments ratio, their physical/chemical nature and microphase separation affect directly on the physical properties, such as glass-transition temperature, mechanical strength, degree of crystallinity, surface topography, etc. So, get enough information about the microstructure of the synthesized TPUs, can play a decisive role on its membrane separation performance and need to be set, precisely^9,10^.

Modeling gas separation membranes give this opportunity to optimize their performance and identify processing bottlenecks. Generally, the mass transport through the dense polymeric membranes obeys the solution-diffusion mechanism. As a penetrant dissolves more, more molecules have a chance to diffuse and then permeate through the membrane. So, predicting the gas solubility and diffusivity are of great importance. Based on the lattice-fluid (LF) model, some researchers^11–13^ have been investigated the prediction capability of the gas solubility and diffusivity, and asserted that this model could calculate reliable results for the polymer membranes by only adjusting characteristic and interaction parameters for sorption and nature of penetrant and polymer media for diffusion.

In order to find a way to model the gas separation properties of copolymers, like TPUs with the approach as mentioned earlier, there is a need to completely know their characterization parameters. The LF model, which is a statistical thermodynamic one, cannot use for polymers without enough information about their Pressure–Volume–Temperature (PVT) data^14^. Furthermore, the general form of Vrentas’ free volume (VSD) model was developed only to predict the diffusion coefficient in liquid/polymer systems that have enough information about the viscoelastic properties of compounds^15,16^. Wang et al.^13^ proposed a way to calculate specific free volume using the LF model that was acceptable for simple polymer structures. In the present study, at first, the characteristic parameters of three types of the TPU membranes with different compositions were determined using a group contribution model to predict the gas solubility, and then the gas diffusion coefficients were calculated using the E-VSD model and free-volume relation proposed by Wang et al.^15,16^. So, the main contribution of this work is to predict the gas separation performance of copolymers with complex nature, especially TPUs, by determining the microstructure properties using analysis of relevant characterization tests and development of existing models.

## Mathematical modeling

The solution-diffusion mechanism is one of the most known theories that can describe the gas permeation behavior of polymeric membranes. At low pressure range, the gas permeability can calculated using multiplication of zero-limit solubility and diffusion coefficient^17^, but at the medium to high pressure ranges, this approach may result in estimating erroneous gas permeabilities. In this work, at steady state condition with assuming no convection terms, an integral relation was extracted which is as follows^18^:

1 $$P_{i} = \frac{1}{{M_{i} p^{u} }}\int_{0}^{{p^{u} }} {(\rho_{p} \frac{{w_{i} }}{p}Z_{i} \times D_{i} )} \, dp$$

where *P*_*i*_*,*, *M*_*i*_, *Z*_*i*_ and *w*_*i*_ are the gas permeability, molecular weight, compressibility factor and weight fraction of dissolved gas-penetrant i, respectively. *p*^*u*^, *ρ*_*p*_ and *D*_*i*_ which were the up-stream pressure, polymer density and diffusion coefficient, respectively. The process of finding Eq. (1) was demonstrated in Eqs. (S1) to (S6) in the supplementary file. The model which was used to calculate compressibility factor, was presented in Eqs. (S7) to (S9).

In this work, a group contribution method was used to calculate the characteristic parameters of TPUs to predict the gas sorption, polymer density and the thermodynamic interaction term and the E-VSD model was extended to calculate the gas diffusion. It has to be pointed out that the following formulation can rearrange for other copolymers with complex structures for which there is no access to their PVT data.

### Formulation of equilibrium sorption isotherms

Despite the multiphase structure of urethane copolymers which have soft and hard segments with different properties, the LF model can be used to model the equilibrium state, as follows^19^:

2 $$\mu_{i}^{F} (T,p) = \mu_{i}^{M} (T,p)$$

where $\mu_{i}^{F}$ and $\mu_{i}^{M}$ are the chemical potential of component *i* in the feed stream and membrane, and *T* and *p* are the operating temperature and pressure, respectively. The chemical potential relation was used as available in the literature^20^.

The LF model suggested an Equation of State (EoS) for the estimating density of both pure gas and polymers, which is expressed as follows^19^:

3 $$\tilde{\rho } = 1 - \exp \left[ { - \frac{{\tilde{\rho }^{2} }}{{\tilde{T}}} - \frac{{\tilde{p}}}{{\tilde{\rho }}} - \left( {1 - \sum\limits_{i = 1}^{n} {\frac{{\phi_{i} }}{{r_{i} }}} } \right)\tilde{\rho }} \right]$$

The other relations and main mixing rules were extracted from the literature^19^.

In order to find the gas sorption into the TPU membranes in a wide range of temperature and pressure, the LF model requires only three parameters. These three parameters are known as characteristic parameters, including $\rho_{{}}^{*}$, $T_{{}}^{*}$, and $p_{{}}^{*}$, which are related to the density of close packed, interaction energy, and cohesive energy (the lattice energy per unit volume), respectively^20^. The characteristic parameters for gaseous compounds directly originated from the literature and are given in the first part of Table 1. To find an estimation of the characteristic parameters for copolymers, Boudouris et al.^21^ suggested a group contribution method, as follows:

4 $$X^{*} = \frac{{X_{0}^{*} + \sum\limits_{k = 1}^{{N_{m} }} {x_{k} \left( {\sum\limits_{i} {N_{i} C_{i} } + \sum\limits_{j} {M_{j} D_{j} } } \right)_{k} } }}{{\sum\limits_{k = 1}^{{N_{m} }} {x_{k} M_{w,k} } }}$$

where $X_{0}^{*}$, *C*_*i*_, and *D*_*j*_ are the *i-*th order group parameters of the Boudouris method, and $N_{i}$ and $M_{j}$ are the number of *i-*th order groups in the structure of copolymer which are comprehensively described in the literature^21,22^. The calculated characteristic parameters for three kinds of TPU copolymers are presented in the second part of Table 1.

**Table 1:** The LF characteristic and interaction parameters for all components.

	T* (K)	p* (MPa)	ρ* (g/cm^3^)	$\updelta _{{{\text{CO}}_{2} }}$	$\updelta _{{{\text{CH}}_{4} }}$	$\updelta _{{{\text{N}}_{2} }}$	References
CO_2_	300	630	1.515	–	–	–	^23^
CH_4_	215	250	0.500	–	–	–	^23^
N_2_	145	160	0.943	–	–	–	^14,24^
TPU-1^1^	658	448	1.100	0.015	− 0.105	− 0.075	This work
TPU-2^2^	564	582	1.125	0.090	− 0.090	0.027	This work
TPU-3^3^	709	600	1.189	0.013	− 0.072	− 0.120	This work

^1^PTMG-2000:1,6-HDI:1,4-BDO (1:3:2).

^2^PPG-2000:TDI:1,4-BDO (1:2:1).

^3^PCL-2000:4,4′-MDI:1,4-BDO (1:3:2).

### E-VSD model for calculation of diffusion coefficients

Vrentas et al.^25^ developed a model based on the free-volume theory to describe the diffusion phenomena in the liquid/polymer systems containing both rubbery and glassy polymers. They considered the Doolittle equation and expanded it by adding some predictable parameters, which were mainly related to the size of the penetrants, physical properties of the polymer, and temperature. According to the VSD model, the diffusion coefficient in the polymeric membrane is calculated by knowing information about the physical and viscoelastic parameters of both penetrant and polymer^26^.

As suggested by Zielinski et al.^27^, the activation energy that fundamentally deals with the needed energy of the polymeric chains to move and provide adequate local free volume, has a value of zero without a reduction in the quality of results. However, for TPUs, with a multiphase structure, the role of activation energy term will be undeniable. So, this term was certainly used for the studied copolymers and was calculated by the following relation as suggested by Zielinski^28^:

5 $$E = E_{0} \times (1 - w_{j} )^{2}$$

To find gas parameters, a method which was suggested by Zielinski, was carried out based on the Dullien relation by adopting the traditional VSD model for the gas molecules in a hypothetical liquid state^28^. The estimated parameters were available in the first part of Table 2 and more explanations were presented in the supplementary file.

**Table 2:** The E-VSD model parameters for the pure gases and copolymers.

	C_1_ (−)	C_2_ (K)	${\widehat{\mathrm{V}}}_{\mathrm{J}}$ (cm^3^ g^−1^)	${\mathrm{M}}_{\mathrm{J}}$ (g mol^−1^)	$\widehat{\mathrm{A}}/\widehat{\mathrm{B}}$(−)	E_0_/R (K)	D_0_ $\times$ 10^4^ (cm^2^ s^−1^)	References
CO_2_	4.08	78.28	0.589	44.01	0.634	–	3.018	^32^
CH_4_	4.43	30.35	1.595	16.04	1	–	0.874	^32^
N_2_	11.8	7.58	0.956	28.01	0.739	–	1.057	^32^
TPU-1	–	–	0.453	167.832	–	1300	–	This work
TPU-2	–	–	0.294	227.803	–	1800	–	This work
TPU-3	–	–	0.441	183.541	–	2000	–	This work

Wang et al.^13^ showed that the VSD model was compatible with the LF model and could predict reliable results. To find free volume, the following relation was utilized as follows^13,29^:

6 $$f - f_{g} = \int_{{T_{g} }}^{T} {\alpha_{f} dT} - 0.5 \times \int_{{p_{0} }}^{p} {\beta_{f} dp} + \int_{0}^{{\phi^{Eq.} }} {\gamma_{f} d\phi }$$

where *f*_*g*_ is the fractional free volume (FFV) at the glass transition point, which is 2.5% as a general constant, *α*_*f*_, *β*_*f*_ and *γ*_*f*_ are the thermal expansion, isothermal compressibility and swelling term, respectively, which are explicitly calculated from Eq. (3) using the following relations^19^^,^^30^:

7 $$\alpha_{f} = - \frac{1}{{\tilde{\rho }}}\left( {\frac{{{\text{d}}\tilde{\rho }}}{{{\text{d}}T}}} \right)_{p} ,\quad \beta_{f} = \frac{1}{{\tilde{\rho }}}\left( {\frac{{{\text{d}}\tilde{\rho }}}{{{\text{d}}p}}} \right)_{T} ,\quad \gamma_{f} = - \frac{1}{{\tilde{\rho }}}\left( {\frac{{{\text{d}}\tilde{\rho }}}{{{\text{d}}\phi }}} \right)_{p,T}$$

With replacing *T*_*g*_ with *T*_*0*_ in Eq. (6), the FFV of the studied copolymers at ambient conditions (T_0_ = 25 °C and p_0_ = 1 bar) was needed. So, this term was calculated using the below relation and adopted with a new method which is completely described and validated in the following sections:

8 $$f(T_{0} ,p_{0} ) = f_{0} = 1 - \frac{{\rho (T_{0} ,p_{0} )}}{{\rho_{c} (p_{0} )}}$$

where *f*_*0*_ and *ρ*_*c*_ is the FFV of copolymer at ambient conditions and density of the fully crystalline region of the copolymer at ambient pressure, respectively.

To increase the accuracy of estimations for the TPU copolymers that has the risk of partial crystallization, the degree of crystallinity was calculated, and corrected the diffusion coefficient using a relation, as follows:

9 $$X_{c} = \left( {\frac{{\rho_{c} }}{\rho }} \right) \times \left( {\frac{{\rho - \rho_{a} }}{{\rho_{c} - \rho_{a} }}} \right)$$

where *X*_*c*_ and *ρ*_*a*_ are the weight fraction of crystallinity and density of the fully amorphous region of the copolymer. The calculated values for the above parameters are gathered in Table 2.

As a result, the final E-VSD equation for estimation of the diffusion coefficient in the TPU membranes is:

10 $$D_{i} = \left( {1 - X_{c} } \right) \times D_{0i} \exp \left( { - \frac{E}{RT}} \right)\exp \left( { - \frac{{\sum\limits_{j = 1}^{n} {w_{j} \frac{{\xi_{iP} }}{{\xi_{jP} }}\hat{V}_{jJ} } }}{{\sum\limits_{j = 1}^{n} {w_{j} \frac{{\hat{V}_{FH,j} }}{{\gamma_{j} }}} }}} \right)$$

where *D*_*i*_ is the self-diffusion coefficient of component i, D_0i_ is the pre-exponential factor, *E* is the activation energy required to break interactions with neighboring molecules of polymer, *ξ*_*iP*_ is the ratio of molar jumping unit of penetrant i to polymer.

The specific free volume of gases and copolymers was calculated using the corrected form of Eq. (6) using Eq. (8), as follows:

11 $$\frac{{\hat{V}_{FH,i} }}{{\gamma_{i} }} = \frac{{\hat{V}_{o} }}{{(2.303 \times C_{1} \times C_{2} )}}\left[ {C_{2} + T - T_{ref} } \right]$$

12 $$\frac{{\hat{V}_{FH,p} }}{{\gamma_{p} }} = \hat{V}_{p} (T,p) \times \left[ {f_{0} + \int_{{T_{0} }}^{T} {\left. {\alpha_{f} } \right|_{{p_{0} ,T}} dT} - 0.5 \times \int_{{p_{0} }}^{p} {\left. {\beta_{f} } \right|_{p,T} dp + \int_{0}^{{\phi^{Eq.} }} {\left. {\gamma_{f} } \right|_{p,T,\phi } d\phi } } } \right]$$

where C_1_ and C_2_ are the viscoelastic parameters of the gas/polymer materials. The relation of the swelling term was presented in Eq. (S11) and (S12) and the relation of expansivity and compressibility factors were extracted from the Wang et al.^13^.

The fully amorphous and fully crystalline densities of the studied copolymers were calculated using the method suggested by Van-Krevelen and Nijenhuis^31^. They asserted that these two parameters could be estimated with an *AARD* of < 5% for at least 35 different polymer/copolymers with complex structures.

Zielinski^28^ suggested a correlation for the copolymer jumping unit that was not completely clear and needed more explanation. Moreover, the later correlation had to be extended for polymers whose *T*_*g*_ was under/near 200 K, because it estimated negative/zero values. So, in this work, for both molecular weight and volume of jumping unit, sparse viewpoint of the previous works was gathered and used to extend the previous VSD model. The two following equations were proposed by Zielinski^28^ and available for other copolymers:

13 $$\frac{1}{{M_{J} }} = \frac{{1 - w_{hs} }}{{0.5 \times M_{ss} }} + \frac{{w_{hs} }}{{1.5 \times M_{hs} }}$$

14 $$V_{J} \left[ {\frac{{cm^{3} }}{gr}} \right] = \frac{{\left[ {(1 - x_{hs} ) \times V_{J} (T_{g,ss} ) + x_{hs} \times V_{J} (T_{g,hs} )} \right]}}{{M_{J} }}$$

where subscripts of *HS* and *SS* are related to the hard segment and soft segment of the TPU structures. The correlated relation for molar volume of jumping unit was extended for copolymers is:

15 $$V_{J} \left[ {\frac{{cm^{3} }}{mol}} \right] = 2.5 \times 10^{ - 3} \times T_{g}^{2} + 7.576 \times 10^{ - 3} \times T_{g} + 82.342,\quad R^{2} = 0.939$$

**Figure 1:**
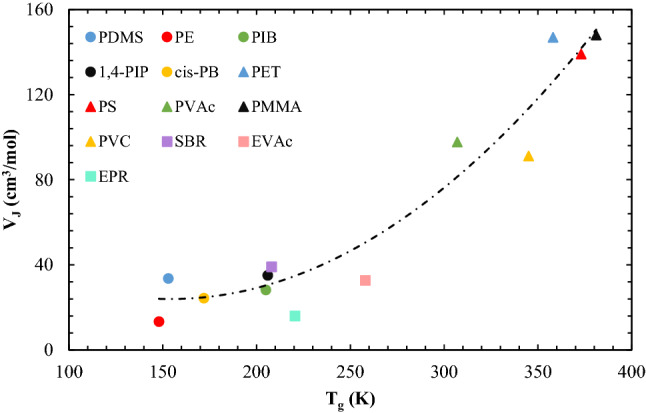
A new correlation for the molar volume of jumping unit of polymers as a function of *T*_*g*_ for polymers in the range of 140 K ≤ *T*_*g*_ ≤ 380 K^28,33^.

The required parameters of the E-VSD model for copolymers are given in the second part of Table 2. As shown in Fig. 1, the correlated equation can be helpful to find the jumping unit volume of copolymers as a function of the glass transition temperature of their soft segment and hard segment, which have different molecular motions.

## Experimental

The microstructure and composition of the TPU copolymers determine the gas separation properties of the TPU membranes to achieve maximum performance^34^. In order to achieve the optimized microstructure, this study proposed a combination of the experimental studies and mathematical modeling that was performed for three different TPU structures. In the following, the material selection and procedure for gaining the required information about the characterization tests were presented.

### TPU membranes preparation

The first TPU sample (TPU-1) was a polymer that was synthesized based on a two-step bulk polymerization method using polytetramethyleneglycol (PTMG, M_n_ = 2000 g.mol^−1^) as a soft segment and a macromolecule of chain-extended 1,6-hexamethylene diisocyanate (1,6-HDI) with 1,4-butanediol (1,4-BDO) as hard segment in the molar ratio of PTMG:HDI:BDO of 1:3:2 according to the procedure presented by Sadeghi et al.^10^. The polyether (PTMG-2000) was purchased from the Arak petrochemical complex (Arak, Iran) and other materials, i.e., 1,6-HDI, 1,4-BDO, dibutyltindilaurate (DBTDL) as the catalyst, dimethylformamide (DMF) as solvent was purchased from Merck Co. (Darmstadt, Germany).

The second TPU sample (TPU-2) was a synthesized polymer that has been previously studied by Ghalei et al.^35^, a copolymer that was based on PPG-2000:1,6-HDI:1,4-BDO with the molar ratio of 1:2:1. The third TPU sample (TPU-3) was provided from Epaflex (185A56, Cassolnovo, PV, Italy) and used as received. The properties of this commercial grade are provided in Table S1 (in the supplementary file).

After drying the TPU-1 and TPU-3 in an air-convection oven at 60 °C for 12 h, a desired amount of the dried granules was dissolved in DMF solvent to obtain a polymer solution with a concentration of 17 wt%. The solution was stirred in an oil bath at 70–80 °C for about 8–12 h to attain a homogenous solution. Then the solution was relaxed at ambient temperature overnight to eliminate the induced residual stresses, due to the stirring process, and cool down the final solution. At last, the desired amount of solution was cast on a flat glass-plate and put in the oven at 80 °C for 12 h to evaporate the solvent and obtain membranes with a thickness of about 40 ± 1 μm. It should be noted that the TPU-2 sample is the same as the TPU-1 in synthesizing, dissolving process, and casting, but they only differ in the type of solvent (DMF for TPU-1 and TPU-3, and chloroform for TPU-2), polyol, and final composition. The thickness of the obtained membranes was 40 ± 3 μm that was measured by a micrometer.

### Characterization of membrane microstructure

The scanning electron microscopy (SEM) technique (AIS2300C, Seron Technologies Inc., South Korea) was used to investigate the surface and cross-section morphology of the membrane samples. To scan the cross-section of the samples, they were floated in the liquid nitrogen and then fractured. After that, all samples were coated with an embedded golden layer. The attenuated total reflectance-Fourier transform infrared (ATR-FTIR) spectroscopy (Nicolet™ iS10™ spectrometer, Madison, WI, USA) was performed between the wavenumber range of 400 cm^−1^ to 4000 cm^−1^. The differential scanning calorimetry (DSC) analysis (Netzsch DSC 214 Polyma, Germany) at the temperature range of − 100 °C to 300 °C with the speed of 10 K min^−1^, and in the N_2_ atmosphere was done. The X-ray diffraction (XRD) (Thermo Scientific™ ARL™ Equinox 3000, USA) with λ of 0.154056 nm was performed at the acceleration voltage of 45 kV. The hydrogen nuclear magnetic resonance (^1^H NMR) analysis (Bruker Ascend™ 850-MHz, USA) was employed to study sequence structure of the TPU membranes. The dynamic mechanical/dynamic mechanical thermal analysis (DMTA) (Netzsch DMA 242 E, Germany) was performed at the temperature range of − 100 °C to 100 °C with the speed rate of 1 K min^−1^. The characterization was done at five different frequencies 0.1, 0.5, 1, 10, and 100 Hz in tensile mode to analyze the viscoelastic behavior of the TPU samples, and also gain information about the available free volume of the membranes. The tensile analysis of the TPU samples was investigated using a tensile testing device (SANTAM STM-1, Tehran, Iran) with a strain rate of 50 mm min^−1^ and a loadcell of 6 Kgf., according to the ASTM D-882 standard.

The TPU-1 and TPU-3 samples were analyzed using a gel permeation chromatography (GPC) system (Waters Breeze, Netherlands), and had the number average molecular weights of 15.39 KDa and 40.83 KDa, respectively. The densities of the TPU-1 and TPU-3 samples, which were determined by a calibrated 50 ml pycnometer at ambient conditions, were 1.038 and 1.130 g cm^−3^, respectively.

### Gas separation performance

A custom-made module employing constant-volume/variable-pressure method was used to study the gas permeation of the TPU membranes for CO_2_, CH_4_, and N_2_ gases. More information about the apparatus for the gas permeation experiments was available in the supplementary file (Figs. S1 and S2).

## Results and discussion

In the following, the required experimental studies are performed, and the theoretical parameters compare to those gained from the characterization experiments. The solubility, diffusivity, and permeability of the studied gases into the TPU copolymers are investigated, and the influence of operating pressure and temperature on the separation properties of three different TPU membranes is investigated. In order to validate the novel formulation for the prediction of the gas solubility, diffusivity, and permeability, the average absolute relative deviation (*AARD*) was considered, which was calculated as follows:

16 $$AARD = \frac{1}{n}\sum\limits_{i = 1}^{n} {\frac{{\left| {X_{i,experimental} - X_{i,theoretical} } \right|}}{{X_{i,experimental} }}}$$

where *n* is the number of experimental data, and *X* represents each gas separation property, i.e., solubility, diffusivity, and permeability.

### Analysis of TPU membranes microstructure

#### SEM analysis

**Figure 2:**
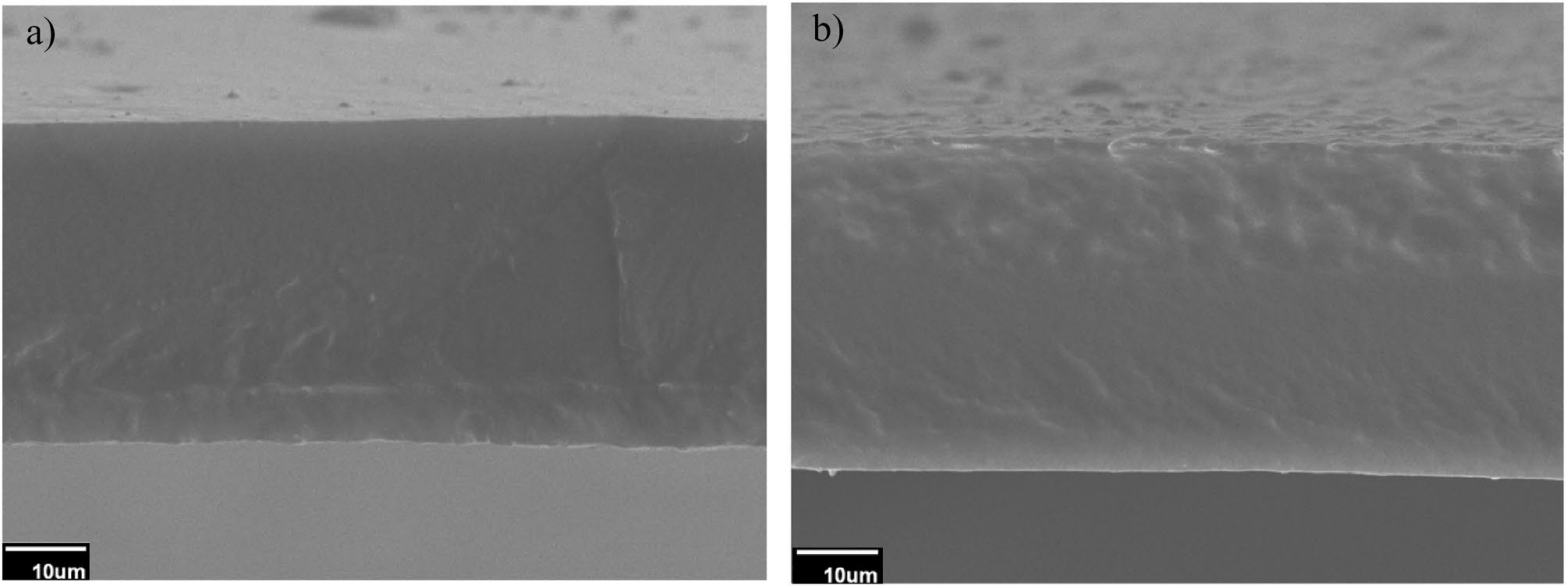
The cross-section SEM image of the TPU membranes: (**a**) TPU-1 and (**b**) TPU-3.

The morphology of the prepared TPU membranes was characterized using the SEM analysis (Figs. 2 and S3). The cross-sectional SEM images show the dense structure of two TPU membrane samples, and in terms of both cross-sectional (Fig. 2a and b) and surface images (Fig. S3), no defect in their structure might disrupt the membrane functionality.

#### ATR-FTIR analysis

**Figure 3:**
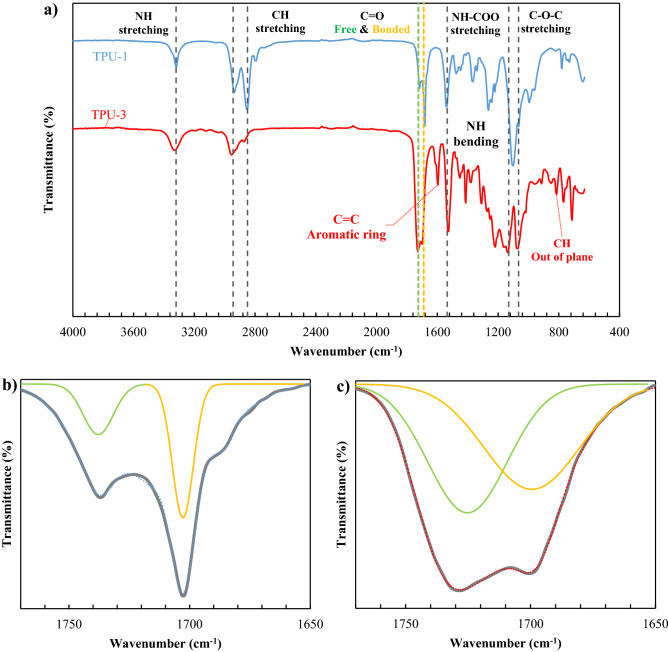
The FTIR spectra of: (**a**) TPU-1 (blue line) and TPU-3 (red-line), and peak analysis around C=O vibration for: (**b**) TPU-1, and (**c**) TPU-3.

The ATR-FTIR spectra of the TPU-1 and TPU-3 are indicated in Fig. 3a. As shown in this figure, the urethane ether linkage (NH-COO) was available for TPU-1 and TPU-3 at 1103 cm^-1^ and 1137 cm^−1^, respectively^10^. The NH stretching peak for TPU-1 and TPU-3 was observed at 3319–3329 cm^−1^, in combination with the absence of a peak at ~ 2230 cm^−1^ (NCO group), should be related to the completion of polymerization^36^. The vibration of C=C in the aromatic ring in combination of CH out of plane bending which was shown at wavenumbers of 1596 and 816 cm^−1^, respectively, was only detected in the spectrum of TPU-3 as an aromatic polyurethane^37^. The most important peaks are those related to bonded and free carbonyl (C=O), which were illustrated in Fig. 3b and c^34,38^. The location of the carbonyl peaks and their integrals are gathered in Table 3. The bonded carbonyl is attributed to hard segment, and the integral of the related peak can be a criterion for measuring the degree of microphase mixing, so the following equations were suggested by some researchers^39,40^:

17 $$X_{b} = \frac{{A_{b} }}{{A_{b} + A_{f} }},\quad w_{hs,FTIR}^{^{\prime}} = \frac{{(1 - X_{b} ) \times w_{hs,theoretical} }}{{(1 - X_{b} ) \times w_{hs,theoretical} + (1 - w_{hs,theoretical} )}}$$

where *X*_*b*_ is the fraction of bonded carbonyl, *A*_*b*_ and *A*_*f*_ are the integrals of bonded and free carbonyl, and *w*_*hs,theoretical*_ is the hard segment fraction of the TPU sample, which was calculated theoretically and can be validated using quantification of the ^1^H NMR analysis. It should be noted that *w′*_*hs,FTIR*_ is the fraction of the hard segment which was dispersed in the soft segment medium and can be used as an index to quantify the degree of microphase mixing. The calculated values for the degree of microphase mixing were 8.05, 12.41, and 19.49 wt.% for the TPU-1, TPU-2, and TPU-3, respectively. These values, in combination with the gas permeation data in the following sections, showed that the content of the hard segment portion was not enough to analyze the results correctly. However, the degree of microphase mixing is the determinative parameter, along with the degree of crystallization.

**Table 3:** The important vibration shifts (cm^−1^) of the FTIR analysis for the TPU.

	Bonded C=O	Free C=O	References
TPU-1	1703 (267)^1^	1737 (87)	This work
TPU-2	1704 (160)	1730 (300)	^35^
TPU-3	1700 (769)	1728 (787)	This work

^1^The integral of peaks in cm^−1^.

#### DSC analysis

**Figure 4:**
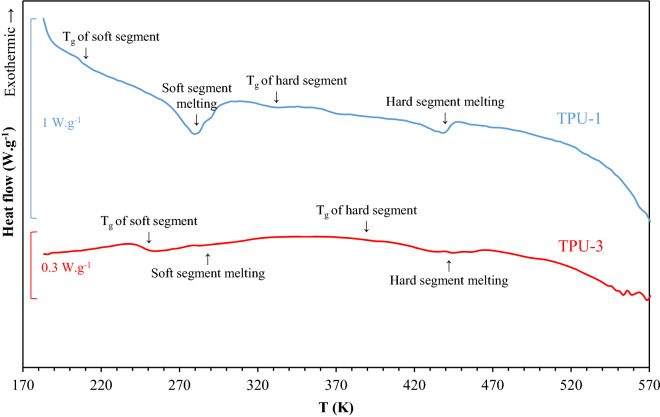
The DSC analysis of the TPU-1 (blue line) and TPU-3 (red line).

The DSC analysis is helpful for determining the thermal transitions and crystallinity of materials. For TPU-1 and TPU-3, the DSC curves are shown in Fig. 4, and the extracted parameters are given in Table 4. In the range of − 100 °C to 300 °C, the first turning point that showed a difference in specific thermal capacity is attributed to the glass transition temperature of the soft segment portion and the second one is for the glass transition of the hard segment portion^10,35,39^. The two endothermic peaks between those related to glass transition, may correspond to breaking the crystalline structures and to the melting point of each portion^41^. The shift of the first transition temperature to higher values may be related to the increment in the degree of microphase mixing^39^. The microphase mixing should be helpful in terms of mechanical strength; however, the decrease in the gas permeation is one of its disadvantages^7^. The interlaminar voids, known as free volume, are at most in the soft segment portion. Nevertheless, the increment in the microphase mixing, with a high degree of H-bonding and the risk of formation of rigid crystalline domains in the hard segment portion, may lead to alter free molecular mobilities of the polyether chains and confine them. This phenomenon creates new linkages between the soft and hard segment molecules in a polyether-based TPU, and increases the interfacial interactions that hinder the gas molecules to dissolve and diffuse, as known for a standalone polyether polymer^42^.

**Table 4:** The transition temperatures (ºC) of the TPU-1 to TPU-3 from the DSC analysis.

	T_g,p-ss_^1^	T_g,ss_	T_g,hs_	T_m,ss_	T_m,hs_	References
TPU-1	− 76.2	− 69.6	59.9	7.4 (32.83)^2^	164.9 (8.41)	This work
TPU-2	− 69.5	− 53.0	47.0	–	–	^38^
TPU-3	− 63.9	− 27.2	120.3	27.8 (0.94)	172.8 (9.40)	This work

^1^Glass transition temperature of soft segment as a standalone polymer.

^2^The integral of peaks in J g^−1^.

From the viewpoint of DSC analysis, the degree of crystallinity and microphase mixing can be calculated using the following equation^39^:

18 $$X_{c} = \frac{{\Delta H_{f} }}{{\Delta H_{f,100\% } }},\quad w_{hs,DSC}^{^{\prime}} = \frac{{T_{g,ss} - T_{g,p - ss} }}{{T_{g,hs} - T_{g,p - ss} }}$$

where *X*_*c*_ is the degree of crystallinity, $\Delta H_{f}$ is the enthalpy of fusion, $\Delta H_{f,100\% }$ is the enthalpy of fusion of a complete crystal, *T*_*g.ss*_ and *T*_*g,hs*_ are the glass transition temperature of the soft and hard segments, and *T*_*g,p-ss*_ is the glass transition temperature of the corresponding polyether polymer. It should be noted that the fusion enthalpy of each sample was extracted from the integral of each endothermic peak. The fusion enthalpy of a complete crystal was calculated using a method suggested by Van-Krevelen and Nijenhuis^31^ with the error limit of 5%. The degree of microphase mixing based on the DSC analysis was 4.84, 14.16, and 19.92 wt.% for the TPU-1, TPU-2, and TPU-3, respectively. These values are in good agreement with those calculated from the FTIR analysis. However, the FTIR analysis gives the maximum available degree of microphase mixing that may lead to a discrepancy in some cases^39^.

#### XRD analysis

**Figure 5:**
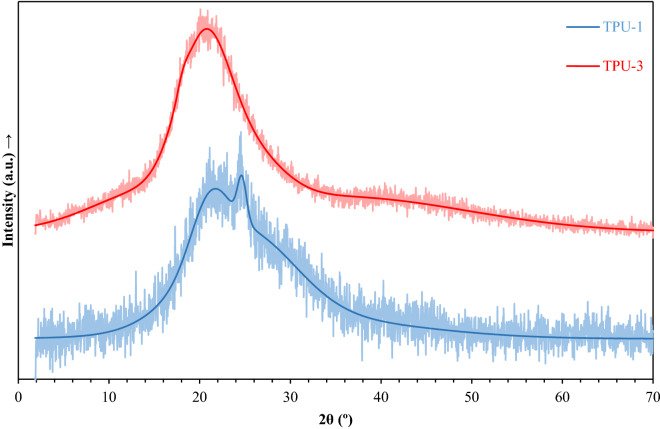
The XRD patterns of the TPU-1 (blue line) and TPU-3 (red line) and their model curves to discrete reflections at appropriate 2θ values.

The XRD patterns of the TPU-1 and TPU-3 membranes are indicated in Fig. 5. As shown in this figure, there are two distinguished reflections at appropriate 2θ values for the TPU-1 sample, the broad one at diffraction angle (2θ) of 21.1° with an integral of 18.33%, and the sharp one at 2θ of 24.6° with an integral of 2.18%^10,43^. The broad one was attributed to small crystallites in the soft segment or diffraction from large crystals, and the sharp one was seen in the HDI-based TPUs^44^. The XRD pattern for the TPU-3 sample showed a broad reflection at 2θ of 20.4° may be attributed to its amorphous structure; however, small crystalline reflections were observed, which could not be easily separated from the large amorphous peaks, so that the DSC analysis would be efficient here. The later analysis investigates each portion separately because each soft or hard segment portion appears in a unique temperature range, so the peaks appeared separately; nevertheless, in the XRD analysis, small crystallite reflections covered by the broad reflection of the amorphous region which both have approximately the same 2θ and cannot be easily separated^10^.

#### DMTA analysis

**Figure 6:**
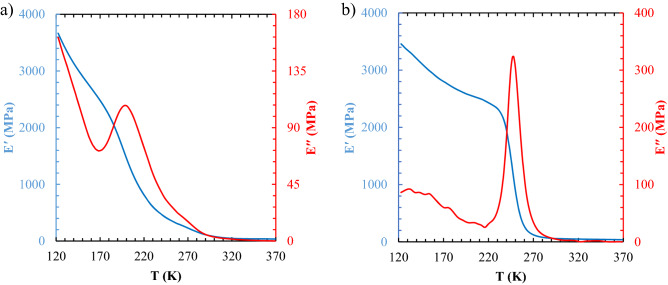
The DMTA analysis of: (**a**) TPU-1 and (**b**) TPU-3 based on tensile mode at a frequency of 1 Hz.

The DMTA analysis of the TPU-1 and TPU-3 at a frequency of 1 Hz is indicated in Fig. 6. As shown in Fig. 6a, the TPU-1 sample in the rubbery region had not a complete plateau behavior because of the high chain mobility of its soft segment (PTMG). On the other hand, in the same region in Fig. 6b, the TPU-3 sample had approximately a plateau behavior. The viscous dissipation modulus of the TPU-1 sample illustrates that its damping behavior near the glass transition region was lower than the TPU-3 sample, by far, so concerning the Young’s modulus of the samples, the TPU-1 was more elastic than TPU-3 at low-range strains, although the toughness and strength (both stress and strain) at break of the TPU-3 sample was more than TPU-1. These results are in consistent with the tensile analysis in Fig. S4. This phenomenon has been observed by some researchers for the TPU-based copolymers^42,45^.

The DMTA, except the capability of studying the glass-transition region, is a tool for linking the viscoelastic properties to available free volume and then the possibility of predicting the gas diffusion^46,47^. Accordingly, a set of DMTA analyses at five different frequencies for the TPU-1 and TPU-3 were carried out, and the two master curves were attained that be useful to find C_1_ and C_2_*,* as shown in Figs. S5 and S6. All information about the DMTA analysis is gathered in Table 5.

**Table 5:** The transition temperature and viscoelastic parameters of the DMTA analysis at a frequency of 1 Hz and T_ref_ = 25 °C.

	T_g,ss_^1^ (°C)	υ_e_ (mol dm^−3^)	C_1_ (−)	C_2_ (K)	References
TPU-1	− 73.60	10.05	66.50	173.35	This work
TPU-2	− 46.00	7.87	–	–	^38^
TPU-3	− 26.40	7.47	100.50	457.85	This work

^1^Based on the inflection of storage modulus (E′) curve.

#### ^1^H NMR analysis

The structure of TPU-1 and TPU-3 were examined using proton analysis, and a quantification technique was chosen to find the exact ingredients, composition, and molecular weight by the ^1^H NMR analysis, and the results are shown in Figs. S7 and S8. Based on the ^1^H NMR analysis, the hard segment portion of TPU-1 and TPU-3 was 24.94 wt% and 29.92 wt%, respectively. The peak analysis was presented in Tables S2 and S3.

Finally, the required parameters from all characterization tests are gathered in Tables 6 and 7, and the experimental results are compared to the theoretical ones based on the investigations, which was performed in the mathematical modeling section. It should be emphasized that there is a good agreement between the obtained T_g,ss_ and T_g,hs_ which were extracted from the DSC and DMTA analyses, so the DSC results are used to calculate the volume of jumping units as a common reference for the TPU-1 to the TPU-3. As to be construed from the results of Table 6, the analysis based on the level of the hard segment is not adequate alone because the soft segment, as the dominant phase in matrix, plays an important role in the bulk properties of the TPU samples^48^. Accordingly, finding out more information about the degree of microphase mixing and interfacial interactions could be efficient to discover the bulk properties of the matrix that may be affected by the dispersed phase. As a result, the formulation could successfully model intrinsic properties of the polymers with proposed structure-based characterization parameters that are needed to gain reliable gas permeability estimations. In the next section, the gas permeation properties of the TPU samples for three different microstructures are studied. The obtained results will be investigated using the substantial evidences that is followed by the experimental section.

**Table 6:** The fundamental parameters in both experimental and theoretical approaches.

	w_hs,model_^1^	w_hs,NMR_^1^	w′_hs,FTIR_^1^	w′_hs,DSC_^1^	T_g,ss_ (°C)	T_g,hs_ (°C)	References
TPU-1	26.18	24.95	8.05	4.84	− 69.6	59.9	This work
TPU-2	18.72	–	12.41	14.16	− 53.0	47.0	^35,38,49^
TPU-3	32.18	29.92	19.49	19.92	− 27.2	120.3	This work

^1^All parameters are in wt%.

**Table 7:** The other parameters of the TPU samples used for modeling.

	f_0_ (%)	X_c,model_ (%)	X_c,DSC_ (%)	X_c,XRD_ (%)	ρ (g cm^−3^)	ρ_pycnometer_ (g cm^−3^)
TPU-1	8.31	21.2	20.5	16.3	1.0413	1.038
TPU-2^35^	8.52	8.5	4.1	–	1.0343	–
TPU-3	8.25	11.3	–	6.1	1.1372	1.130

### Gas separation performance

The assessment of the mathematical modeling to predict reliable gas permeabilities, and the gas permeation experiments for the TPU-1 and TPU-3 membranes were performed, simultaneously. After that, the relationship between the gas permeation and microstructure properties will be investigated.

#### Pressure effects on gas permeation properties of membranes

**Figure 7:**
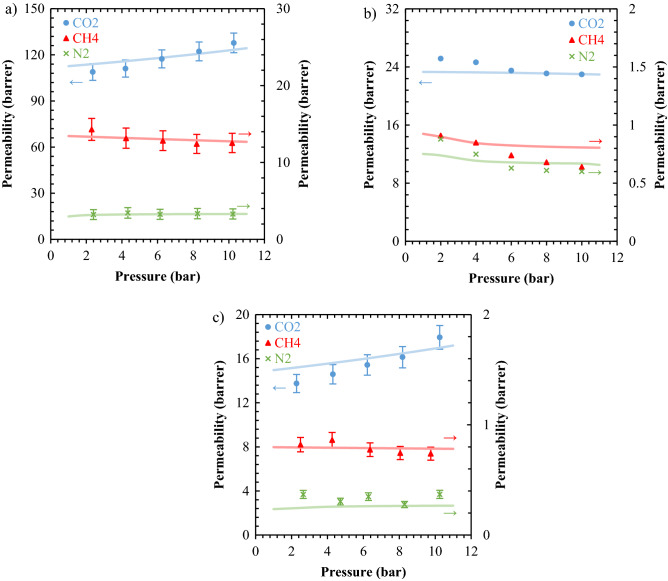
The experimental (dots) and theoretical (lines) gas permeabilities as a function of the operating pressure: (**a**) TPU-1, (**b**) TPU-2^35^, and (**c**) TPU-3.

Based on the solution-diffusion mechanism, the gas solubility and diffusivity determine the net value of the gas permeability, so getting knowledge about the factors affecting both solubility and diffusivity is of great importance. Along with the experimental investigation, the ability of mathematical models to predict gas permeabilities was studied. In Fig. 7, the gas permeability of three types of TPU membranes as a function of pressure is presented. It can be seen that the CO_2_ permeability of the TPU-1 and TPU-3 membranes enhances as the operating pressure increases, while the CO_2_ permeability of the TPU-2 membrane decreases. As noted by many researchers, the permeability of the glassy polymers is a descending function of pressure^50^, but this occurs vice versa for the rubbery polymers^51^. Copolymers, like TPUs, which are phase-separated, may behave unpredictably and need to investigate precisely. According to our findings from the characterization of the TPU membranes, the differences in the pressure dependency of the gas permeabilities could be ascribed to the differences in the microstructure of the samples, so the main aspects are: (i) the amount of the hard segment, (ii) the degree of microphase mixing, (iii) the degree of crystallinity, and iv) the FFV of the copolymer. The hard segment in the TPU copolymers is quite glassy and have not enough chain mobility to create the void space needed for the gas diffusion^52^. Moreover, this portion with a high degree of the structure order, may crystallize easily in some cases (HDI as isocyanate) and can be impermeable^10,40^. Nevertheless, the soft segment has more chain mobility and free voids than the hard segment, and most importantly, it has a significant number of ether groups that is desired to achieve higher degrees of gas sorption. For TPU-1, TPU-2 and TPU-3, the degree of crystallinity, which was 21.16, 8.50, and 11.34 wt%, respectively, showed the samples had impermeable regions. Furthermore, this parameter was in consistent with the FFV at ambient condition, as shown in Table 7 as f_0_. Any factor that leads to microphase mixing, such as synthesis method, composition, reaction conditions, casting process, etc., can dominate the properties of the hard segment over the soft segment and affect the properties of the final TPU membrane^53^. In the TPU-1, the permeability of all gases, except CH_4_, had ascending behavior versus pressure. In the meantime, CO_2_ was more permeable than other gases because of more condensability and higher affinity with the ether groups in the polymer matrix. For the TPU-2, all three gases permeated as a descending function of pressure, and as before, the permeability of CO_2_ was higher than the other gases. Because of the high degree of microphase mixing in the TPU-2 (14.16 wt%) the decrement of permeability versus pressure would be forecastable, concerning TPU-1 with 4.84 wt% of microphase mixing. In the analysis of the gas permeability results, the percentage of the hard segment cannot be relied on alone, but the percentage of microphase mixing along with the degree of crystallinity is an influential and decisive factor. The high microphase mixing forces the gas molecules to dissolve and diffuse less than before, because the dispersion of the potentially crystallized domains, which confines chain mobility of the soft segment, is in favor of this happening. For TPU-3, except CO_2_ gas, all the other gases permeated as a descending function of pressure. The lower level of the gas permeability for TPU-2 was because of its high microphase mixing, 19.92 wt%. It showed that all synthesized TPU structures, with controlled synthesis process based on gaining a lower degree of microphase mixing, are suitable in terms of gas permeation. Moreover, the studied polyester-based TPU, with high degree of crystallinity and strong covalent bonds, is not consistent with the gas permeation performance.

### The diffusion prediction using experimental viscoelastic parameters

**Figure 8:**
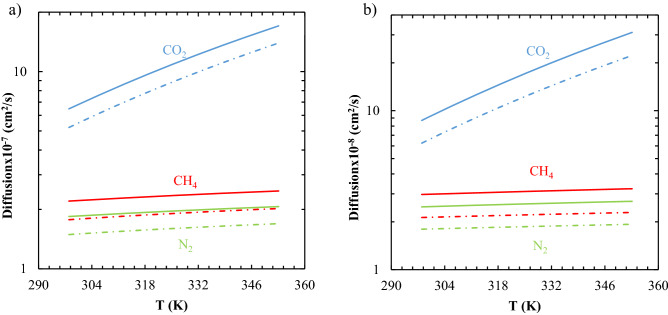
The prediction of the gas diffusion coefficients at *p* = 0.1 MPa based on the modeling (lines) and viscoelastic parameters of the DMTA analysis (dashed lines): (**a**) TPU-1, and (**b**) TPU-3.

The descending behavior of the permeability versus the operating pressure originates mainly from the descending behavior of the gas sorption coefficient because the effect of pressure on the changes of the gas diffusion coefficient is mostly negligible^8,13^. The latter case might be challenging and need to be more obviously investigated. For this purpose, by performing a set of DMTA analyses for the TPU-1 and TPU-3, the C_1_ and C_2_ parameters were estimated from the obtained master curve using an optimization algorithm by Netzsch proteus software as shown in Figs. S3 and S4. The viscoelastic parameters were used directly to predict the free volume of the samples and to determine the diffusion coefficients. The results are shown in Fig. 8, and the capability of the extended diffusion model to predict reliable output was distinct. It should be noted that as the tensile mode of DMTA analysis considered dynamic-mechanical properties of solid samples to find viscoelastic criteria, this may lead to present viscoelastic parameters higher than those calculated from the shear analysis, which is done in liquid/melt state^46,47^. So, it led to estimate specific free volume, and then self-diffusion, less than estimated by the LF model, as shown in Fig. 8a and b.

**Figure 9:**
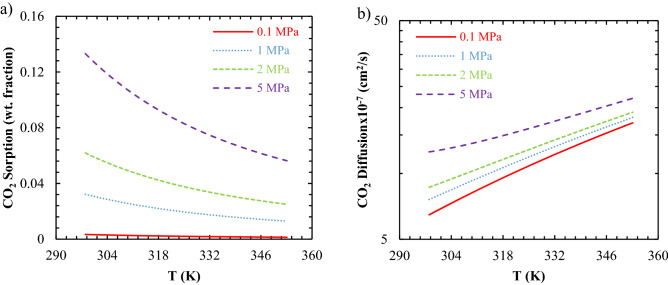
The prediction of the pressure-induced (0.1 to 5 MPa): (**a**) sorption and (**b**) diffusion as a function of temperature (25–80 °C) for the CO_2_/TPU-1 system.

The E-VSD model can predict gas diffusion as a function of temperature and pressure. It can be seen that the CO_2_ diffusion coefficient in the TPU-1 membrane enhanced by increasing both operating temperature and pressure (Fig. 9a). As shown in Fig. 9b, the gas sorption also increased by changing the pressure from the ambient pressure (0.1 MPa) to 30 MPa. More available dissolved gas into the membrane led to higher gas diffusion; however, as the pressure increased, the membranes were going to compact and led to a decrement in the diffusion. These two unlike phenomena occurred simultaneously, and as inferred from Fig. 9a, the increment in the dissolved gas overcame the compaction effects, and the diffusion coefficients increased by increasing pressure (up to 10 MPa), as observed in Li et al.^54^. The different curvature of the predicted diffusion at pressures greater than 10 MPa might be due to the altering behavior of the dissolved gas versus pressure at the middle range pressures (up to 30 bars), which was previously illustrated for the solubility of CO_2_/PDMS pair^14^.

## Conclusion

In this work, the gas separation performance of three different TPU membranes was investigated experimentally and theoretically, and the relation between the gas separation properties of the TPU membranes with their microstructures was studied. The experimental section was helped the theoretical one to enrich the required structural parameters and capable of extending for polymers with complex structures, like TPUs. To enable traditional models for the prediction of the gas separation performance, accurately, the degree of crystallinity and activation energy terms were added to the fundamental diffusion relation. The FFV relation of polymers was successfully corrected using the concept of impermeable crystalline domains and calculated uniquely for each microstructure. The polymer density was predicted using a new set of characteristic parameters for each TPU sample and led to a precise calculation of the degree of crystallinity and FFV at ambient conditions. The T_g_ of all samples were agreed with each other in terms of both DSC and DMTA tests. The hard segment fraction and degree of crystallinity, estimated using theoretical methods, were compared to the ^1^H NMR, and DSC tests, and results with AARD of < 8% and < 40%, respectively, were obtained. Moreover, the calculation of the gas solubility and diffusivity were accomplished using the characteristic parameters and improved diffusion relation. As a result, the gas permeabilities reliably predicted with respect to the experimental data (AARD < 5%). Using the viscoelastic parameters from the DMTA analysis, the free volume and diffusivity were calculated underestimate concerning the theoretical results (AARD < 54%) due to the complex nature of the TPU samples. The analysis of the microstructures of the TPU membrane samples revealed that the type of polyol and diisocyanate significantly affect the physicochemical properties of the obtained membranes as well as their separation properties. For example, the TPU-1 membrane containing PTMG and HDI had a crystallinity degree of 21.1%, while the crystallinity degree of the TPU-2 membrane containing PPG and TDI was 8.5%. The existence of a polyol with side group (TPU-2) in the structure of TPU provides microstructural irregularity and hinder increment of crystalline domains. The degree of microphase mixing determined using the DSC and FTIR analyses was in the order of TPU-1 < TPU-2 < TPU-3 and inconsistent with the gas permeation results. The ^1^H NMR analysis showed that the hard segment portion of the TPU-1 and TPU-3 is 24.94 wt.% and 29.92 wt.%, respectively. It was observed that the TPU-1 membrane had higher gas solubilities and permeabilities due to its lower degree of microphase mixing and higher degree of crystallinity. The microstructure analysis of the TPU membranes indicated that the content of the hard segment is not enough to analyze the separation performance of the copolymer membranes correctly, and the degree of microphase mixing along with the degree of crystallinity is the determinative parameter. Furthermore, the E-VSD model enables to predict the gas diffusion through the TPU membranes as a function of temperature and pressure, and it was found that the CO_2_ gas sorption and diffusion in the TPU-1 membrane increased by enhancing both operating temperature and pressure. It implies that more available dissolved gas into the membrane leads to higher gas diffusion, although, higher operating pressure results in the membrane compaction. Finally, it can be concluded that the microstructure analysis of the complex copolymer membranes, such as TPUs, can predict their gas separation performance and could be employed as an effective tool to design membranes with appropriate properties for the separation of a given gas mixture.

## Supplementary Information

Supplementary Information.

## Data Availability

The data that support the findings of this study are available from the corresponding author upon reasonable request.

## Latin symbols

*AARD* %: Absolute average relative deviation (%)
$\widehat{A}/\widehat{B}$: Aspect ratio of gaseous penetrants (−)
*C*_*i*_: First order parameters of *i*-th group in the repeating unit
*D*_*j*_: Second order parameters of *i*-th group in the repeating unit
*D*_*i*_: Self-diffusion coefficient of component *i*
*D*_*oi*_: Pre-exponential factor of E-VSD model (cm^2^ s^−^^1^)
*E*_0_: Activation energy of polymeric-chains diffusion (j mol^−^^1^)
*f*_0_: Fractional free volume (FFV) at ambient condition (T = 25 °C, *p* = 1 bar)
*M*_*j*_: Number of second order parameters of *i*-th group in the repeating unit
*N*_*i*_: Number of first order parameters of *i*-th group in the repeating unit
*N*_*p*_: Number of penetrants (−)
*N*_*m*_: Number of blocks in a copolymer (−)
*P*_*i*_: Permeability of gas component *i* (barrer)
*p*: Pressure (bar)
*p*^***^_*i*_: Characteristic pressure of component *i* (MPa)
*∆p*^***^_*ij*_: Interaction parameter of LF model for binary ij components (MPa)
*Q*: Thermodynamic interaction parameters (−)
*R*: Universal gas constant (j mol^−^^1^ K^−^^1^)
*T*: Absolute temperature (°C)
*T*^***^_*i*_: Characteristic temperature of component *i* (°C)
*T*_*g,p-ss*_: Glass transition temperature of standalone polyether (°C)
*T*_*g,ss*_: Glass transition temperature of the soft segment of copolymer (°C)
*T*_*g,hs*_: Glass transition temperature of the hard segment of copolymer (°C)
*V*^*id*^: Ideal gas volume (cm^3^ g^−^^1^)
${\widehat{V}}_{FH.i}$: Specific free volume of component *i* (cm^3^ g^−^^1^)
${\widehat{V}}_{iJ}$: Volume of molecular jumping unit of component *i* (cm^3^ g^−^^1^)
$\widehat{V}(T,p)$: Mass volume of polymer at T and p (cm^3^ g^−^^1^)
*w*_*i*_: Weight fraction of dissolved gas (gr/gr)
*w*_*hs*_: Mass fraction of hard segment portion
*w′*_*hs*_: Mass fraction of hard segment that is mixed in soft segment portion
*x*_*hs*_: Mole fraction of hard segment portion
*X*^***^: Represents for each characteristic parameter in the Boudouris relation
*z*: Compressibility factor (−)

## Greek symbols

*α*: Thermal expansion of polymer in the rubbery state (K^−^^1^)
*β*: Compressibility factor of polymer in the rubbery state (bar^−^^1^)
*γ*_*i*_: Overlap factor of component i (−)
*γ*_*f*_: Swelling factor (−)
*δ*_*ij*_: Interaction parameter of LF model (−)
*μ*_*i*_: Chemical potential of component *i* (j mol^−^^1^)
*υ*^***^_*i*_: Characteristic volume of component *i* (cm^3^ mol^−^^1^)
*υ*^***^: Characteristic volume of mixture (cm^3^ mol^−^^1^)
*ρ*_*i*_: Density of pure component *i* (g cm^−^^3^)
*ρ*^***^_*i*_: Characteristic density of component *i* (g cm^−^^3^)
*φ*_*i*_: Volume fraction of component *i*
*φ*_*i*_^*Eq*^^*.*^: Volume fraction of component *i* at equilibrium state
*ξ*_*ij*_: Interaction parameter of E-VSD model (−)

## Subscripts

*i*: Pure gas component *i*
*j*: Pure gas component *j*
*p*: Pure polymer component *p*
