# Supplementary material for: A thermodynamic study on relationship between gas separation properties and microstructure of polyurethane membranes
Source: Sci Rep. 2023 Apr 13;13:6038. doi: 10.1038/s41598-023-32908-7 (PMC10102001; doi:10.1038/s41598-023-32908-7)
Supplement: Supplementary file 1 — Supplementary Information. [file 41598_2023_32908_MOESM1_ESM.docx]

**Supplementary data for**

**Study on relationship between gas separation properties and microstructure of polyurethane membranes: a combination of theoretical and experimental techniques**

Mohammad Sajad Sepehri Sadeghian, Ahmadreza Raisi^*^

Department of Chemical Engineering, Amirkabir University of Technology (Tehran Polytechnic), Hafez Ave., P.O. Box 15875-4413, Tehran, Iran

^*^Author for correspondence: Phone: (9821) 64543125, Fax: (9821) 66405847,

E-mail: [raisia@aut.ac.ir](mailto:raisia@aut.ac.ir)

**S1. The model parameters**

The mass balance relation in the Cartesian coordinates is as follows:

| $\rho\left(\frac{\partial w_{i}}{\partial t}+v.\nabla w_{i}\right)=-\nabla.j_{i}+r_{a}$ | )S1( |
| --- | --- |

At steady state condition with neglecting convection terms through dense membrane structure and no mass production, the following relation for unidirectional penetration of component i is resulted:

| $-\frac{dj_{i}}{dx}=0\to j_{i}=-\rho D\frac{dw_{i}}{dx}=\text{constant}$ | )S2( |
| --- | --- |

So, the final integral relation for calculation of mass flux is:

| $j_{i}=\frac{1}{l}\int_{0}^{w_{i}^{Eq.}} \rho D_{i}Q_{i}dw_{i}$ | )S3( |
| --- | --- |

The thermodynamic interaction parameter can be calculated with Eq. (S10) and (S12).

On the other hand, there is a relationship between mass flux and permeability as follows:

| $j_{i}\equiv P_{i}\frac{\Delta p}{l}$ | )S4( |
| --- | --- |

With assuming ^1^:

| $Q_{i}dw_{i}=w_{i}\frac{Z_{i}}{p_{i}}dp_{i}$ | )S5( |
| --- | --- |

Consequently, the final relationship for permeability is as follows:

| $P_{i}=\frac{1}{M_{i}p^{u}}\int_{0}^{p^{u}} \rho\frac{w_{i}}{p_{i}}Z_{i}\times D_{i}dp_{i}$ | )S6( |
| --- | --- |

The compressibility factor of CO_2_, CH_4_ and N_2_ gases were calculated using the Peng-Robinson model which was as follows:

| $Z=\frac{pv}{RT}=\frac{v}{v-b}-\frac{v}{RT}\left(\frac{a\alpha}{v^{2}+2bv-b^{2}}\right)$ | )S7( |
| --- | --- |
| $a=0.45724\frac{R^{2}T_{c}^{2}}{P_{c}}$ , $b=0.07780\frac{RT_{c}}{P_{c}}$ | (S8) |
| $\alpha=(1+(0.37464+1.54226\omega-0.26992\omega^{2})(1-T_{r}^{0.5}))^{2}$ | (S9) |

The thermodynamic interaction and swelling terms which were calculated using the LF model is as follows ^2^:

|  | (S10) |
| --- | --- |
| $\gamma_{i}=-\frac{F_{1}}{1/(1-\tilde{\rho})+1/(\tilde{\rho}r)-2/\tilde{T}}$ | (S11) |
|  | (S12) |

To calculate the gas penetrants parameters for the VSD model, a set of viscosity and density of the prior gas molecules in their liquid state was extracted from the literature and then using the Dullien relation and by fitting the experimental data, the gas parameters were calculated ^3–6^. It should be noted that i and j subscripts are known as gas and polymer components.

**S2. The physical properties of TPU-3 sample**

The physical properties of the TPU-3 sample are gathered in Table S1. The information is originated from EPAMOULD catalogue, polyester 100 series, 185A56 ([www.Epaflex.it](http://www.Epaflex.it)). This material was processed by injection molding, had no blooming agent with an opaque and yellowish appearance.

**Table S1**: The physical properties of the TPU-3 sample, based on the available information of Epaflex Company.

| Typical properties | Unit | Value | Method |
| --- | --- | --- | --- |
| Density | g.cm^-3^ | 1.20 | ISO 1183 |
| Hardness Shore A | - | 85 | ISO 868 |
| Tensile strength | MPa | 35 | EN 12803 |
| Stress @ 100% elongation | MPa | 5.5 | EN 12803 |
| Elongation at break | % | 590 | EN 12803 |
| Tear strength | kN.m^-1^ | 67 | ISO 34 |
| Abrasion resistance | mm^3^ | 50 | EN 12770 |
| Glass-transition temperature | ºC | -25 | DMA^*^ |
| ^*^ Based on the maximum loss modulus. | | | |

**S3. The gas permeation apparatus**

The gas permeation performance of the TPU samples were analyzed at room temperature by a constant-volume/variable-pressure technique. The single gas permeation of CO_2_, CH_4_, and N_2_ gases was investigated with a transmembrane pressure of 2 to 10 bar. The gas permeation through a prepared membrane was accomplished first for N_2_, then for CH_4_, and finally for CO_2_. The gas permeability of the membranes was defined as follows ^7^:

| $P=\frac{273.15\times10^{10}\times V\times l}{760\times A\times T\times\left(76p_{0}\right)}\left(\frac{dp}{dt}\right)$ | (S13) |
| --- | --- |

Where *P* is the gas permeability (Barrer), *A* is the membrane area (12.54 cm^2^), *T* is the operating temperature (K), *V* is the downstream volume of the membrane (10.08 cm^3^), *l* is the membrane thickness (μm), *p_0_* is the feed pressure (bar), and (*dp/dt*) is the rate of pressure increase in the down-stream (bar.s^-1^). The downstream side of the membrane module was under vacuum before starting each gas permeation experiment. Each gas permeation test was repeated three times, and the membranes were allowed to soak in each gas for 2500 s. A schematic representation of the experimental apparatus is shown in Fig. S1.


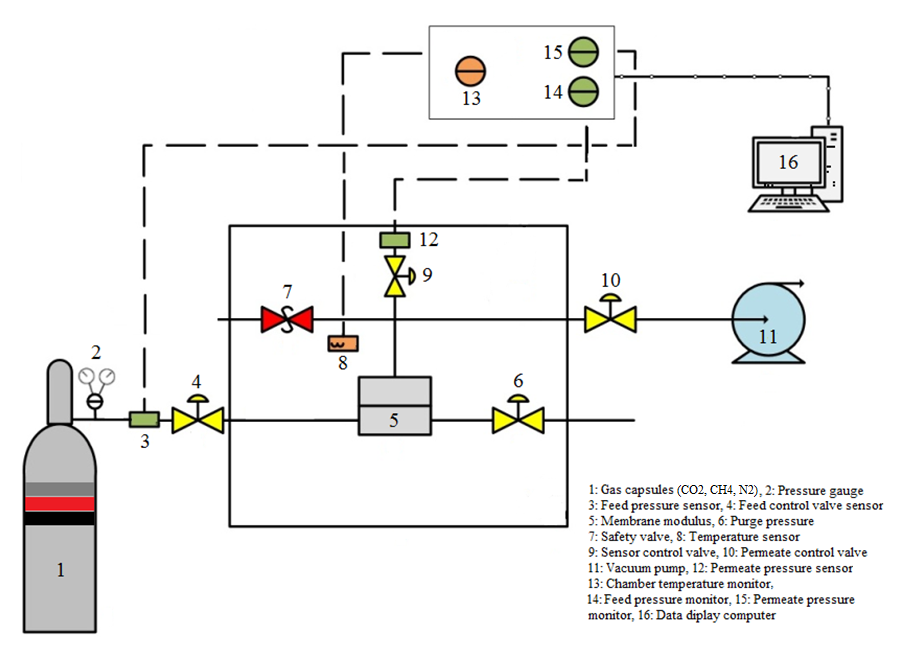


**Fig. S1**: The schematic diagram of the single gas-permeation apparatus.

An image of membrane module is shown in Fig. S2. This custom-made apparatus was manufactured from steel 316 because of preventing any corrosion in presence of CO_2_ and air humidity. The feed was moved through fittings with no leak (checked for every test set) and permeate through membrane. The permeated gas molecules accumulated in the dead-end permeate chamber and the detector recorded the required data. The O-ring (outer diameter of 43 mm with thickness of 1.5 mm) was made from Viton B and changed for every test set. The accuracy of pressure sensor in the permeate side is 200 mbar.


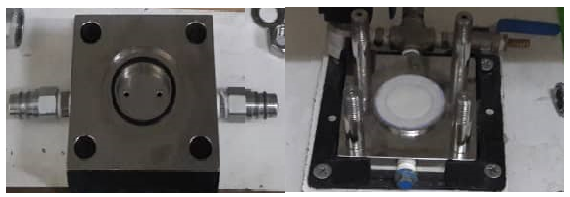


**Fig. S2**: The membrane module.

**S4. The SEM analysis**

The surface SEM images of the TPU-1 and TPU-3 samples are presented in Fig. S3. As shown, there was no distinct defect in the surface images. Furthermore, the observed surface distortion was most likely due to rubber-like behavior of the soft segment portion, as the dominant phase, in each sample which was probably made during film casting process.

| 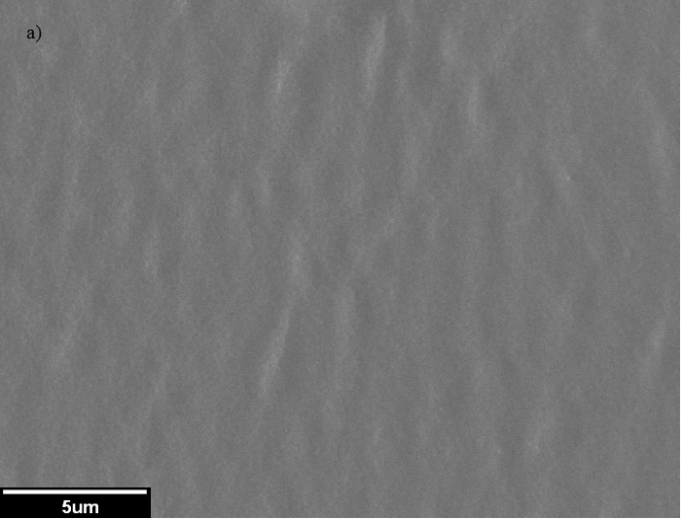 | 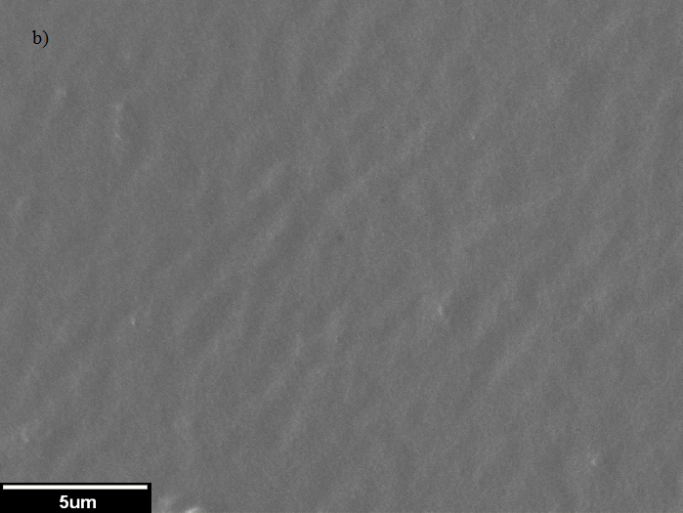 |
| --- | --- |

**Fig. S3**: The surface SEM image of the PU membranes: (a) TPU-1 and (b) TPU-3.

**S5. The tensile analysis**

The tensile behavior and properties of the TPU-1 and TPU-3 samples are presented in Fig. S4. The width of the samples was 10 mm. The thickness of TPU-1 and TPU-3 were 0.035 and 0.04 mm, respectively.

|  |
| --- |
|  |

**Fig. S4**: The tensile test analysis of the TPU-1 (blue line) and TPU-3 (red line) samples both in the full strain-range (a) and in the elastic-to-plastic strain-range (b).

**S6. The DMTA analysis**

The master curve of the DMTA analysis of the TPU-1 and TPU-3 samples is shown in Figs. S5 and S6, respectively, and the viscoelastic parameters (T_ref_=25ºC) were extracted by the Netzsch proteus software.


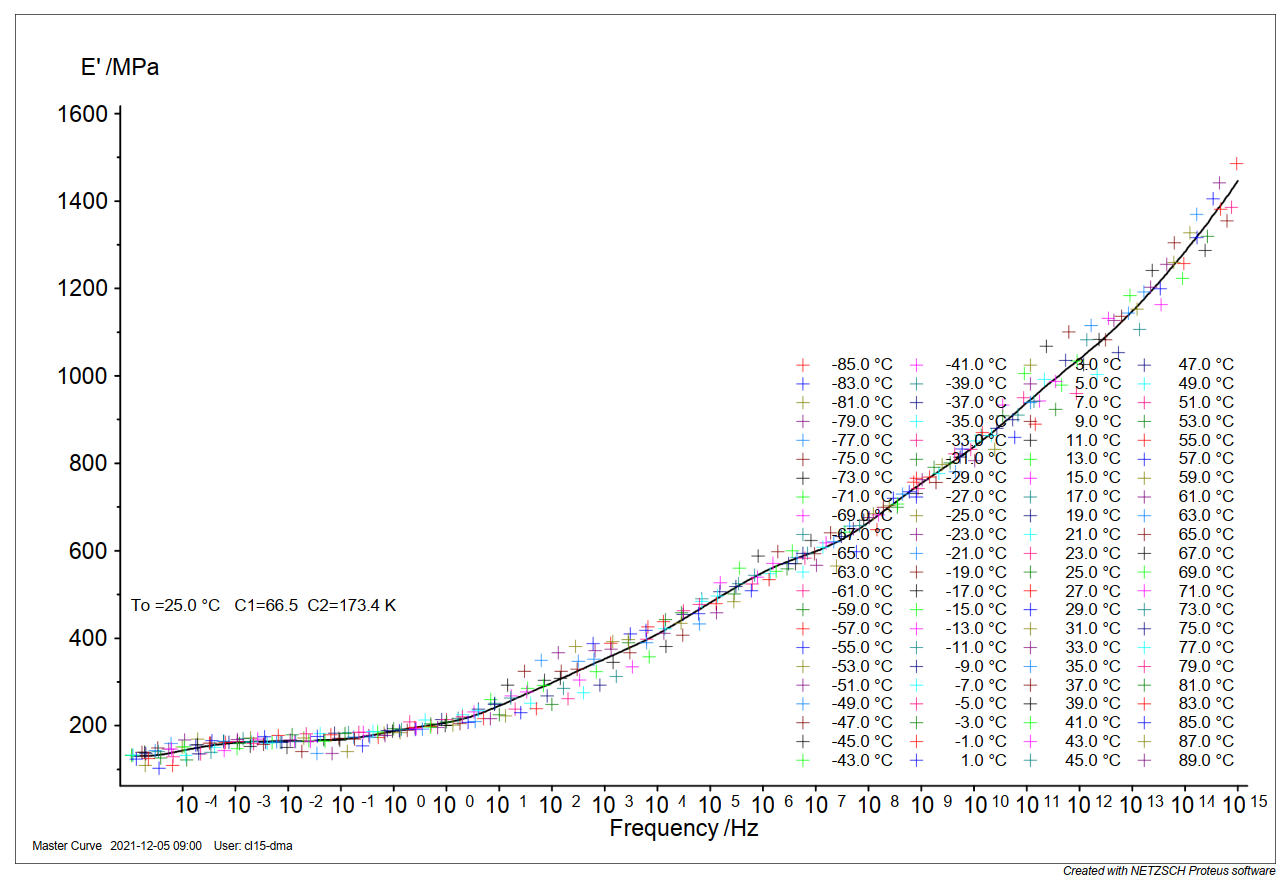


**Fig. S5**: The master curve of DMTA analysis of the TPU-1 sample.


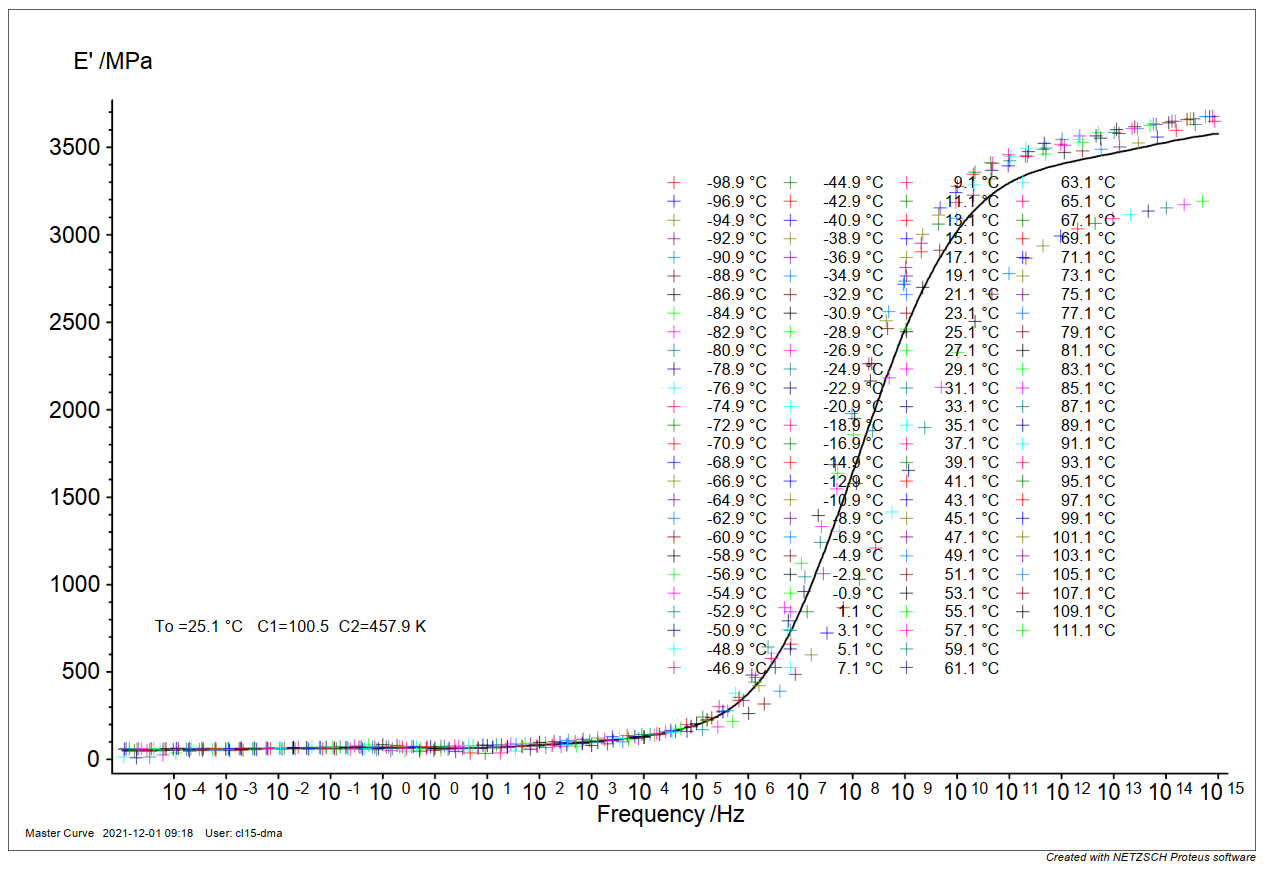


**Fig. S6**: The master curve of DMTA analysis of the TPU-3 sample.

**S7. The ^1^H NMR analysis**

The ^1^H NMR and peak analysis of the TPU-1 and TPU-3 are shown in Figs. S7 and S8, and Tables S1 and S2 using Peyravi et al. and Tang et al. studies ^8,9^. It should be noted that for the TPU-1 sample, there is not a difference between protons of polyether and chain extender, so the first row of the Table S1 was related to both soft segment and hard segment. The number of repeating unit of the TPU-1 and TPU-3 was calculated as 5.571 and 13.548, respectively ^10^.


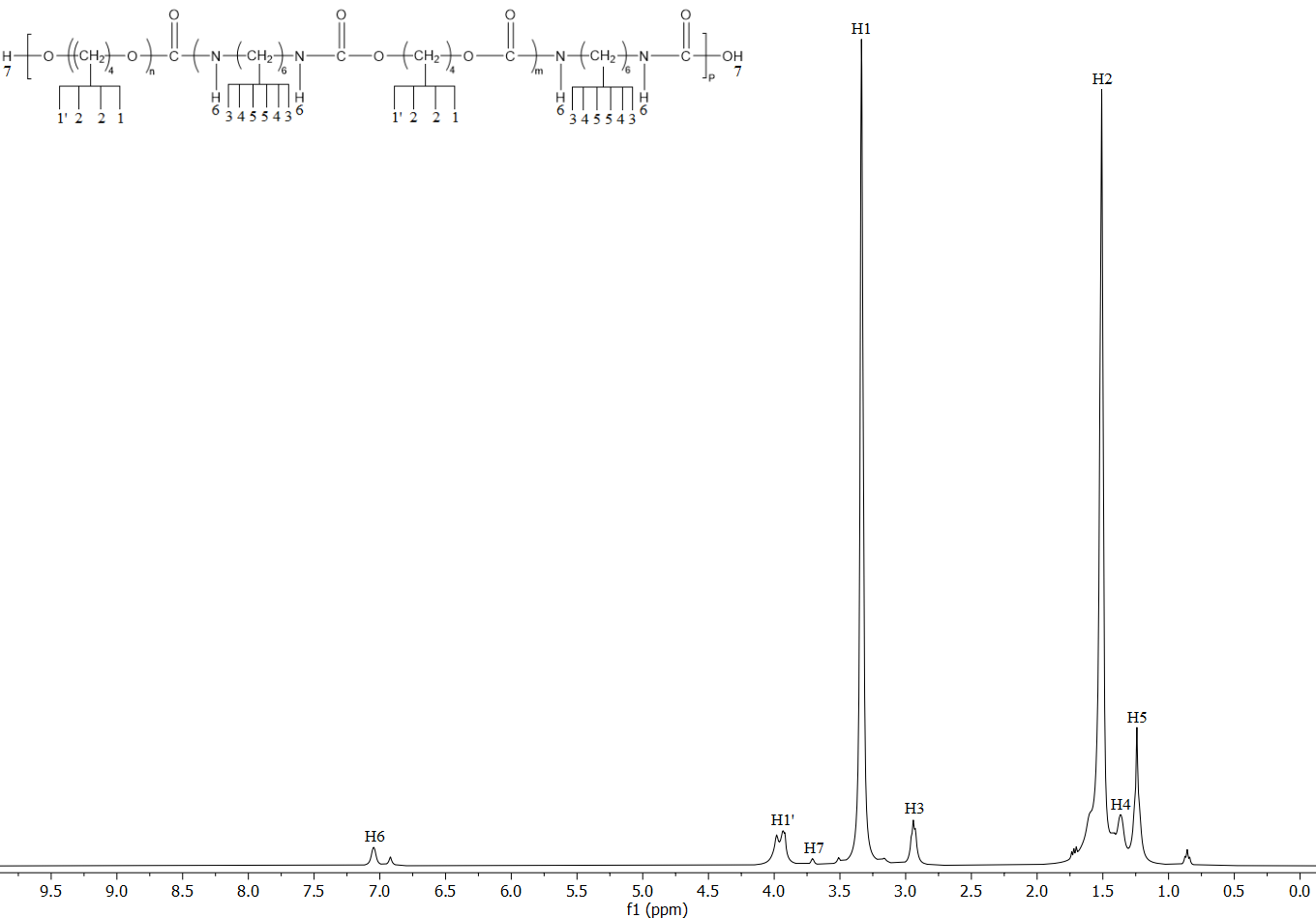


**Fig. S7**: The ^1^H NMR analysis of the TPU-1 sample.

**Table S2**: The ^1^H NMR peak discretization of the TPU-1 sample.

| Number | Moiety | Shift (ppm) | Multiplicity | Protons | Peak area |
| --- | --- | --- | --- | --- | --- |
| 1+1′+2 | CH_2_CH_2_-O | 1.51-3.51-3.34 | Multiplet | 4 | 86.98 |
| 3+4+5+6 | CH_2_CH_2_CH_2_CH_2_-NH-C=O | 1.24-1.36-2.94-7.05 | Multiplet | 9 | 13.56 |
| 7 | OH | 3.71 | Singlet | 1 | 0.31 |

**
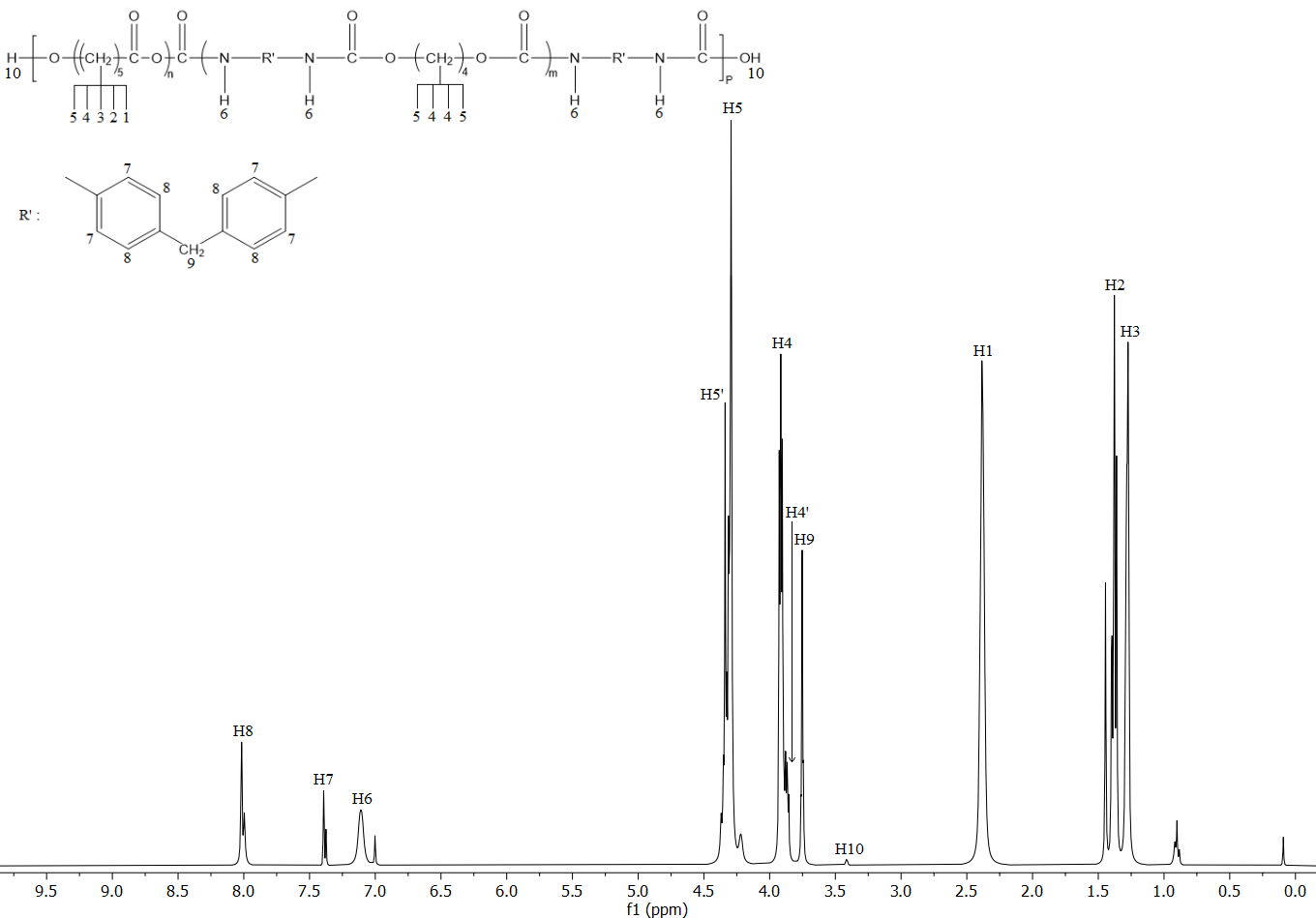
**

**Fig. S8**: The ^1^H NMR analysis of the TPU-3 sample.

**Table S3:** The ^1^H NMR peak discretization of the TPU-3 sample.

| Number | Moiety | Shift (ppm) | Multiplicity | Protons | Peak area |
| --- | --- | --- | --- | --- | --- |
| 1+2+3 | CH_2_CH_2_CH_2_-C=O | 1.28-1.38-2.39 | Mulitplet | 6 | 48.26 |
| 4+5 | CH_2_CH_2_-O | 3.93-4.31 | Multiplet | 3 | 25.07 |
| 4′+5′ | CH_2_CH_2_-O | 3.87-4.34 | Multiplet | 4 | 6.95 |
| 6 | NH-C=O | 7.11 | Singlet | 1 | 2.51 |
| 7+8 | Ar-C-CH-CH | 7.39-8.02 | Quartet | 2 | 10.34 |
| 9 | Ar-CH_2_ | 3.75 | Singlet | 2 | 2.48 |
| 10 | OH | 3.41 | Singlet | 1 | 0.088 |

**References**

1. Minelli, M. & Sarti, G. C. Permeability and diffusivity of CO2 in glassy polymers with and without plasticization. *J. Memb. Sci.* **435**, 176–185 (2013).

2. Sanchez, I. C. & Lacombe, R. H. Statistical Thermodynamics of Polymer Solutions. *Macromolecules* **11**, 1145–1156 (1978).

3. Zielinski, J. M. Free-volume parameter estimations for polymer / solvent diffusion coefficient predictions. (The Pennsylvania State University, 1992).

4. Herreman, W., Grevendonk, W. & De Bock, A. Shear viscosity measurements of liquid carbon dioxide. *J. Chem. Phys.* **53**, 185–189 (1970).

5. Hellemans, J. Zink, H., Van Paemel, O. The viscosity of liquid nitrogen and liquid oxygen along isotherms as a function of pressure. *Physica* **47**, 45–57 (1970).

6. Hellemans, J., Zink, H. & Van Paemel, O. The viscosity of liquid argon and liquid methane along isotherms as a function of pressure. *Physica* **46**, 395–410 (1970).

7. Zarshenas, K., Raisi, A. & Aroujalian, A. Mixed matrix membrane of nano-zeolite NaX/poly (ether-block-amide) for gas separation applications. *J. Memb. Sci.* **510**, 270–283 (2016).

8. Peyravi M., B. A. A. Study on the Synthesis of Poly(ether-block-amide) Copolymer Based on Nylon6 and Poly(ethylene oxide) with Various Block Lengths. *J. Appl. Polym. Sci.* (2010). doi:10.1002/app.32358

9. Tang, Q. & Gao, K. Structure analysis of polyether-based thermoplastic polyurethane elastomers by FTIR, 1H NMR and 13C NMR. *Int. J. Polym. Anal. Charact.* **22**, 569–574 (2017).

10. Izunobi, J. U. & Higginbotham, C. L. Polymer molecular weight analysis by 1H NMR spectroscopy. *J. Chem. Educ.* **88**, 1098–1104 (2011).
